# Asymptotic behaviour and optimal word size for exact and approximate word matches between random sequences

**DOI:** 10.1186/1471-2105-7-S5-S21

**Published:** 2006-12-18

**Authors:** Sylvain Forêt, Miriam R Kantorovitz, Conrad J Burden

**Affiliations:** 1 Mathematical Sciences Institute, Australian National University,Canberra, ACT 0200, Australia; 2 Department of Mathematics, University of Illinois, Urbana, IL 61801, USA

## Abstract

**Background:**

The number of *k*-words shared between two sequences is a simple and effcient alignment-free sequence comparison method. This statistic, *D*_2_, has been used for the clustering of EST sequences. Sequence comparison based on *D*_2 _is extremely fast, its runtime is proportional to the size of the sequences under scrutiny, whereas alignment-based comparisons have a worst-case run time proportional to the square of the size. Recent studies have tackled the rigorous study of the statistical distribution of *D*_2_, and asymptotic regimes have been derived. The distribution of approximate *k*-word matches has also been studied.

**Results:**

We have computed the *D*_2 _optimal word size for various sequence lengths, and for both perfect and approximate word matches. Kolmogorov-Smirnov tests show *D*_2 _to have a compound Poisson distribution at the optimal word size for small sequence lengths (below 400 letters) and a normal distribution at the optimal word size for large sequence lengths (above 1600 letters). We find that the *D*_2 _statistic outperforms BLAST in the comparison of artificially evolved sequences, and performs similarly to other methods based on exact word matches. These results obtained with randomly generated sequences are also valid for sequences derived from human genomic DNA.

**Conclusion:**

We have characterized the distribution of the *D*_2 _statistic at optimal word sizes. We find that the best trade-off between computational efficiency and accuracy is obtained with exact word matches. Given that our numerical tests have not included sequence shuffling, transposition or splicing, the improvements over existing methods reported here underestimate that expected in real sequences. Because of the linear run time and of the known normal asymptotic behavior, *D*_2_-based methods are most appropriate for large genomic sequences.

## Background

The overwhelming amount of molecular data generated by the sequencing of whole genomes and EST libraries has triggered the development of numerous sequence comparison algorithms, aimed at detecting related sequences and at quantifying this relatedness. BLAST [1], FASTA [2] and other related algorithm are arguably the most popular programs for sequence comparison. These algorithms rely on the local alignment of the sequences under scrutiny and assume conservation of contiguity between homologous segments. This assumption, however, is often violated. Discontinuity can occur, for example, when spliced transcripts are matched to genomic sequences, when ESTs or cDNAs from different splice variants are compared or when genomic sequences are aligned that have undergone genome shuffling. Other alignment-based algorithms that do not assume conservation of contiguity, such as BLAT [3] or SIM4 [4], can compute scores, percentage similarity, but do not assess statistical significance.

Several types of alignment-free sequence comparison algorithms, reviewed in [5], can circumvent this problem. Among these alignment-free methods, techniques based on the number of *k*-words shared between two sequences, also known as the *D*_2 _statistic, are particularly noteworthy due to the speed of their implementation, their sensitivity and selectivity [6]. They have been extensively used to structure large collections of ESTs into clusters of similar sequences [7-9].

The rigorous study of the statistical distribution of *D*_2 _began with the computation of bounds of *D*_2 _variance and the characterization of asymptotic distributional regimes [10]. These results have been refined in a recent study [11]. Other studies [12,13] have focussed on a generalization of *D*_2_, the number of approximate *k*-word matches between two sequences, , where *t *is the number of mismatches per word. Bounds on thevariance and asymptotic distribution of  have been determined [13].

The current paper summarizes the theoretical knowledge on the distribution of *D*_2 _and . The optimal word sizes of these statistics in a variety of conditions were computed, and the distributional regimes at optimal word size were deduced. Finally, the accuracy of  as a measure of sequence similarity was compared with other measures using random sequences and sequences evolved from human genomic DNA.

## Results and Discussion

### Word matches measures

#### Exact matches

The theory developed for the number of exact word matches between two sequences is widely applicable to any kind of sequence with the only constraint that these are made of independent and identically distributed (i.i.d.) letters. Given an alphabet  of *d *letters, let **A **= *A*_1_*A*_2 _⋯ *A_*n *_*be a sequence of *n *i.i.d. letters of . Let *f_a _*be the probability of a letter taking the value *a*, and *p_k _*= .

*D*_2 _is defined as the number of *k*-words matches between two sequences **A **and **B**, and can be expressed



where *Y*_(*i, j*) _is the *k*-word match indicator variable starting at position *i *in **A **and *j *in **B**, and the index set is

*I *= {(*i*, *j*) : 1 ≤ *i *≤ , 1 ≤ *j *}

where, for convenience, (*n - k *+ 1) and (*m - k *+ 1) are written  and  respectively.

*D*_2 _can also be thought of as an inner product of the word count vectors. Let  = {**w_1_, w_2_, ⋯, w**_*n*_} be the set of all *k*-words on . For w ∈ , let  be the number of times the letter **w **appears in sequence **A**. The count vector for that sequence is . *D*_2 _can thus also be expressed as



The mean of *D*_2 _can be easily computed (e.g. [14])



When the letters of the alphabet are uniformly distributed, that is, *f*_*a *_= , for all *a *∈ *A*.



Lower and upper bounds for the variance of *D*_2 _were computed in [10], and an exact formula for the variance in the case of uniform letter distribution was given in [11]. Limiting distributions of *D*_2 _when *n *and *k *get large were derived in [10], namely (a) when *k*/*n *> 2, and *E*[*D*_2_] is not too small, *D*_2 _has a compound Poisson [15] asymptotic behaviour, and (b) when *k*/*n *< 1/6, and the letter distribution is non-uniform, *D*_2 _has a normal asymptotic behaviour. Numerical simulations in [10] showed that these theoretical bounds are not tight. The uniform letter distribution case was studied in [11], where it was proved that, for large enough *k*, the *D*_2 _statistic is approximately normal as *n *gets large.

#### Approximate matches

The theory for the number of approximate matches between two sequences has been developed in the more restricted framework of strand symmetric Bernoulli text [12,13]. In this framework, the letters are i.i.d. with frequencies





where *η *is the perturbation parameter, with -1 ≥ η ≥ 1. The distance between two words *x *and *y *of length *k *is defined as the number of character mismatches between *x *and *y *and is written *δ*(*x, y*). When *δ*(*x, y*) ≥ *t, x *is said to be a *t*-neighbour of *y*. If **A **is a strand symmetric Bernoulli text and **w **is a known word of the same length, *n*, as **A**, then the probability distribution of the distance *δ*(**A, w**) is:

Pr(*δ*(**A, w**) = *t*) =*g_t_*(*n,η,c*)

where *g_t _*is a perturbed binomial distribution [12], *c *is the *GC *count in **w **and *η *is the perturbation parameter of **A**. The cumulative distribution function of the distance is then



The  statistic is defined to be the number of *t*-neighbours of *k*-words between sequences **A **and **B**, and can be expressed as



where  is the indicator variable of *t*-neighbourhood for the *k*-words starting at position *i *in **A **and *j *in **B**. The expectation of  can be expressed in terms of the perturbed binomial distribution [12]



Upper and lower bounds for the variance of  were computed in [13], and the following limit deduced: for large *n*,  is asymptotically normal when *k *= *α*(*n*) + *C *where 0 ≥ *α *< and *C *is a constant. When *t *= 0, this result is an improvement on the *α *= 1/6 result for perfect matches [10] reported above. Numerical simulations in [13] suggest that this asymptotic behaviour occurs for *α *as high as 2.

**Figure 1:**
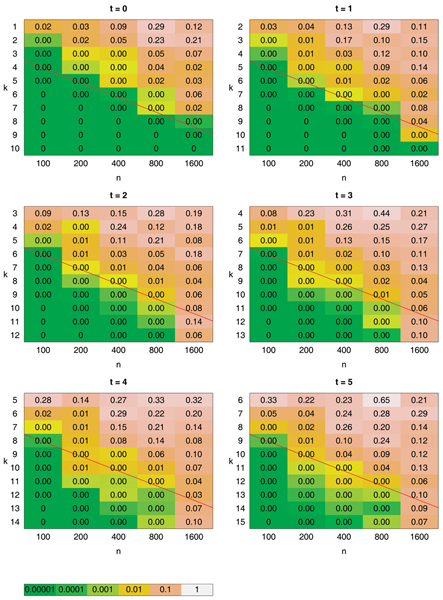
**Kolmogorov-Smirnov p values for non-uniform ****compared with normal**. (adapted from [13]) The letter distribution is *f_A _*= *f_T_=*, *f_G _*= *f_C _*= , and the diagonal red line in each table is *k *= 2*n *+ const.

The results of the numerical simulations comparing the distribution of  to a normal distribution, for various word sizes, sequences sizes and number of mismatches, in the case of non-uniform letters distribution, are shown in figure 1. Similar tables comparing *D*_2 _to the normal and compound Poisson distribution, in the case of exact word matches, for uniform and non-uniform letters distribution, can be found in [10].

### Optimal word sizes

Our first goal is to characterize the distribution regime of  when the word size is optimal. We write  the optimal word size for sequences of size *n *and *t *mismatches. Numerical simulations were carried out to determine  for various sequence lengths *n *= 2*^x ^*× 10^2 ^with *x *= 1, 2, 3, 4, 5, and for different numbers of mismatches *t *= 0, 1, 2, 3, 4, 5. A summary of these simulations is given in table 1 and the detailed simulation results can be found in additional files 1 to 9. Sequences with a non-uniform letter distribution were used, with nucleotide frequencies *f_A _*= *f_T _*= , *f_G _*= *f_C _*= . Similar compositional biases are observed in several sequenced genomes, such as the honey bee *Apis mellifera*, the roundworm *Caenorhabditis elegans *or the zebra fish *Danio rerio*. The optimal word size was also computed in the uniform case for *t *= 0, 3, with very similar outcomes. We will therefore focus our discussion on the non-uniform case.

Regardless of the number of mismatches, the optimal word size is quite stable when the lengths of the sequences vary across the range of sizes under consideration. We only noticed a slight decrease in  for sequences smaller than 400 letters (see table 1). The behaviour of , however, can traverse different distribution regimes when the sequence length varies and the word size is fixed.

When our data for perfect word matches are compared to tables 1 and 3 in [10], it appears that the distribution of *D*_2 _at optimal word size is approximately a compound Poisson distribution when sequences are 400 letters long or smaller, and is approximately normal when sequences lengths are larger than 1600 letters.

In the case of approximate word matches, it can be extrapolated from figure 1 that the distribution of  becomes normal for sequences larger than 1600 letters. The approximate behaviour of , when *t *> 0 and *k*/*n *> 2, however, is unknown. The distribution regime of  at optimal word size for smaller sequences could therefore not be characterized.

### Accuracy of  measures

**Figure 2:**
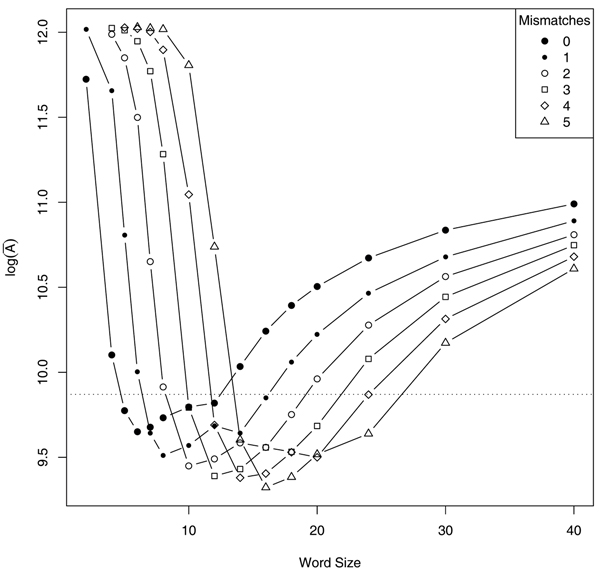
**Effect of the number of mismatches on the accuracy of **. The curves show the accuracy of the  statistics according to the word length and for various mismatches per word. In this simulation, sequence sizes of 600 letters were used. The dotted line shows the results obtained with BLAST.

A previous study [16] compared the accuracy of various dissimilarity measures based on the number of words shared between 2 sequences. These authors give the values of the Spearman's rank statistic, *A *(see methods), obtained with these measures and compare them with that obtained using either BLAST or the Hamming distance. Similarly, we compared the efficiency of  for different numbers of mismatches, *t *= 0,1, 2, 3, 4, 5 (figure 2). We used the same sequence size (600) as inthe above-cited study so as to allow comparison of the performance of  with the measures assessed in that study. *A *for BLAST was computed based on the bit scores obtained with the default settings.

Overall, the  measures provide an accuracy similar to the dissimilarity measures computed in [16], with log (*A*) ranging from 9.3 to 9.6. This is better than BLAST whose log (*A*) is close to 9.9. It is noteworthy that, at optimal word size, the  statistic gives better results when the number of mismatches allowed per word increases.

### Application to non-iid sequences

In real biological sequences, letters are not independent and identically distributed. To test the applicability of our results to biological sequences, we conducted similar simulations, but instead of randomly generated sequences of known composition, we used sequences sampled from the human genome and made them evolve according to the K80 [17] model of nucleotide substitutions. The results are summarized in additional file 9. These results are quite encouraging in many respects. First, the optimal word sizes for these sequences are the same as or very close to the ones predicted with random sequences in the previous sections. The values of log (*A*) for near-optimum word sizes are lower than those computed previously, suggesting that similarity measures based on word counts may be more accurate on real sequences than on randomly generated sequences. Finally, the accuracy of the  measures near optimal word size are consistently better than BLAST bit scores obtained with the default settings.

## Conclusion

The  statistic has different distribution regimes depending on the word size *k*, the sequences lengths *n*, the number of mismatches *t *and the sequence composition *f_*ATGC*_*. We computed the optimal word size for various combinations of these parameters that influence the distribution of . For sequences smaller than 400 letters and when no mismatches are allowed, the distribution of  is close to a compound Poisson. In the case of sequence larger than 1600 letters, the distribution of  is approximately normal. When estimating the significance of sequence similarity using  at its optimal word size, the null distribution would thus have to be adjusted according to the size of the sequences. It is worth noting that the size of typical ESTs or whole genome shotgun sequencing traces (500–800 letters) is in the transition region between these two limiting distributions.

We have also shown that the accuracy of the  statistic is similar to that of other similarity measures based on the number of words shared between two sequences. In particular, allowing for mismatches between words increases the accuracy of the . This improvement, however, comes at a high computation cost. The algorithmic complexity of  is *o*(*kn*), when *t *= 0, however, when *t *> 0, the worst case complexity is *o*(*knm*). In our simulations,  was more accurate than BLAST (see figure 2). We expect this difference to be accentuated on real sequences where shuffling and transposition occurs, breaking the contiguity generally assumed by alignment-based sequence comparison algorithms.

The assumption of i.i.d. distributed letters made to ease the characterization of the  distribution may not hold in real sequences. This may have an impact on the assessment of the statistical significance of the number of words shared between two sequences. The optimal word sizes computed in this study, however, seem to be valid for real sequences evolved under a relatively realistic model of nucleotides substitution.

Taken together, the statistical theory, the algorithmic complexity and the simulation results suggest that the best application of the  statistics would be the identification of similarities between large genomic sequences using exact word matches. Comparison of such sequences using alignment-based methods is computationally expensive, since these algorithms typically have a *o*(*n*^2^) complexity, hence the development of heuristics speeding up these comparisons, such as BLAT [3] or MEGABLAST [18]. The linear (*o*(*n*)) nature of the  algorithm and the normal asymptotic behavior of , when sequences are large compared to word size, would allow sequence similarity to be assessed in a rigorous statistical framework, with significant improvement in run time.

Finally, the known asymptotic behavior of the  statistic could be used to improve the assessment of significant matches during the initial (search) stage of existing alignment algorithms such as BLAST or BLAT.

The use of  in the context of biological sequences is not limited to the sequence comparison discussed in this paper. It has been proposed that it could be used to choose discriminative microarray probes [12]. Other possible applications may include the detection of transcription factor binding sites, microRNAs and dsRNA targets.

## Methods

### Word size optimization

The optimization of the word size, for a given sequence length and number of mismatches, was carried out according to a method similar to that introduced in [16]. In brief, a family of sequences was generated by first creating a random mother sequence. 100 sons were then derived by mutating γ% of the mother sequence, where γ = 1, 2, . . ., 100%. Only point mutations were used: substitution, insertion and deletion of a single letter. The  statistic between the mother and each son were computed. Two rankings of the sons were then produced, one based on , and another based on γ. The accuracy of -based sequence comparison was estimated by looking at the discrepancy between these two rankings by means of the Spearman's rank statistic *A*. The optimal word size is that for which *A *is minimal. For each condition, the data presented here are the average of *A *for 100 to 400 families.

Similar simulations were also carried out by randomly selecting a mother sequence from the human genome chromosome 1, version NCBI36.40 (available from ). Sons where then derived according to a K80 [17] model of evolution.

A program written in ANSI C was written to compute the *spearmanRS *statistic. Data were post-processed using the R environment. The simulations were carried out on a Debian Gnu/Linux desktop computer. The source code is available from our *k*-words website [19].

### Fit to the normal distribution

Kolmogorov-Smirnov *p*-values [20] for the standardized statistic  compared with the standard normal distribution for sample sizes of 2,500 sequence pairs were computed. The results are shown in figure 1. In general, samples which are a close approximation to the normal distribution have *p*-values distributed uniformly in the interval [0, 1], whereas samples which are a poor approximation have small *p*-values.

## Authors contributions

SF drafted most of the manuscript and performed the numerical simulations. MK made all the theoretical calculations. CB conceived of and coordinated the project and drafted parts of the manuscript. All authors read and approved the final manuscript.

## Supplementary Material

Additional file 1 Word size optimization for exact word matches, uniform sequences Click here for file

Additional file 2 Word size optimization for exact word matches, non-uniform sequences Click here for file

Additional file 3 Word size optimization for approximate word matches with 1 mismatch, non-uniform sequences Click here for file

Additional file 4 Word size optimization for approximate word matches with 2 mismatch, non-uniform sequences Click here for file

Additional file 5 Word size optimization for approximate word matches with 3 mismatch, uniform sequences Click here for file

Additional file 6 Word size optimization for approximate word matches with 3 mismatch, non-uniform sequences Click here for file

Additional file 7 Word size optimization for approximate word matches with 4 mismatch, non-uniform sequences Click here for file

Additional file 8 Word size optimization for approximate word matches with 5 mismatch, non-uniform sequences Click here for file

Additional file 9 Word size optimization for exact and approximate word matches, sequences evolving according to a Kimura80 substitution model. The dotted lines show the results obtained with blast. Click here for file

## Figures and Tables

**Table 1:** Optimal word sizes. Optimal word sizes for various sequence lengths and numbers of mismatches.

Mismatches	Sequence length				
	200	400	800	1600	3200
0	6	7	7	7	7
1	8	10	10	10	10
2	10	12	12	12	12
3	12	14	14	14	14
4	14	16	16	16	16
5	16	18	18	18	18
